# Short- and long-term effects of anti-CD20 treatment on B cell ontogeny in bone marrow of patients with rheumatoid arthritis

**DOI:** 10.1186/ar2789

**Published:** 2009-08-17

**Authors:** Maria Rehnberg, Sylvie Amu, Andrej Tarkowski, Maria I Bokarewa, Mikael Brisslert

**Affiliations:** 1 Department of Rheumatology and Inflammation Research, Sahlgrenska Academy at University of Gothenburg, Guldhedsgatan 10A, 413 46 Gothenburg, Sweden

## Abstract

**Introduction:**

In the present study we evaluated changes in the B cell phenotype in peripheral blood and bone marrow (BM) of patients with rheumatoid arthritis (RA) following anti-CD20 treatment using rituximab.

**Methods:**

Blood and BM samples were obtained from 37 patients with RA prior to rituximab treatment. Ten of these patients were resampled 1 month following rituximab, 14 patients after 3 months and the remaining 13 patients were included in the long-term follow up. B cell populations were characterized by CD27/IgD/CD38/CD24 expression.

**Results:**

One and three months following rituximab BM retained up to 30% of B cells while circulation was totally depleted of B cells. Analysis of the remaining BM B cells showed prevalence of immature and/or transitional B cells (CD38^++^CD24^++^) and CD27^+^IgD^- ^memory cells, while IgD^+ ^cells were completely depleted. A significant reduction of CD27^+ ^cells in BM and in circulation was observed long after rituximab treatment (mean 22 months), while levels of naive B cells in BM and in circulation were increased. The levels of rheumatoid factor decline after rituximab treatment but returned to baseline levels at the time of retreatment.

**Conclusions:**

Anti-CD20 treatment achieves a depletion of IgD^+ ^B cells shortly after the treatment. At the long term follow up, a reduction of CD27^+ ^B cells was observed in blood and BM. The prolonged inability to up-regulate CD27 may inhibit the renewal of memory B cells. This reduction of CD27^+ ^B cells does not prevent autoantibody production suggesting that mechanisms regulating the formation of auto reactive clones are not disrupted by rituximab.

## Introduction

B cells are important players in the pathogenesis of rheumatoid arthritis (RA) [1,2]. The products of autoreactive B cells, rheumatoid factor (RF) and recently recognised antibodies against citrullinated peptides are the established markers of severe RA leading to progressive joint destruction, early disability and mortality [3,4]. Rituximab, a chimeric monoclonal antibody targeting B cells expressing CD20 antigen, is a prevalent and highly efficient strategy for the treatment of RA when the disease is non-responsive to conventional disease-modifying anti-rheumatic drugs (DMARDs) and anti-TNFα blockade. Treatment with rituximab results in the prolonged alleviation of clinical symptoms of RA and reduction of inflammation [5-8]. Alleviation of clinical symptoms occurs simultaneously with a reduction of autoantibody levels, while the levels of antimicrobial antibodies as well as total levels of immunoglobulins (Ig) do not change [9,10]. These observations suggested a selective depletion of a B cell population with potential impact on the pathogenesis of RA.

The expression of CD20 antigen is restricted to the B cell population. It occurs at the early pre-B cell stage of development and remains through out all stages of B cell maturation being down-regulated on plasma blasts/plasma cells. The initial stages of B cell development take place in bone marrow (BM) where autoreactive immature B cells are eliminated by negative selection. The maturation of B cells in BM is characterised by surface expression of IgD and IgM. The mature B cells that have not been antigen activated (also called antigen naïve) leave BM and migrate via peripheral blood (PB) to secondary lymphoid tissue such as the spleen and lymph nodes. Here they change/switch the expression pattern of Ig from IgD and IgM to IgG, IgA and IgE. The switch of Ig classes indicates the formation of antigen-specific memory B cells. By the expression of CD27 and IgD, developmental stages of B cells may be identified, as immature B cells (CD27^-^IgD^-^), naïve B cells (CD27^-^IgD^+^), un-switched memory B cells (CD27^+^IgD^+^) and switched memory cells (CD27^+^IgD^-^). The population of switched memory B cells may contain even plasma blasts/cells [11-13]. The expression of CD38 in combination with IgD may also be used to determine the maturation status on B cells. Due to bi-polar expression of CD38 its intermediate expression characterizes early pre-B cells and transitional cells, and its high expression characterizes end-stage differentiated plasma blasts/cells. To gain more information about the maturation stages of the B cell population, expression of CD24 and CD10 is usually added [14-25].

The exact subpopulation of B cells targeted and eliminated by rituximab remains uncertain. Several studies investigated the effects of rituximab with respect to its effect on leukocytes in different body compartments and showed an efficient depletion of B cells in circulation, while the number of plasma cells was not changed [26-32]. A reduction of B cells short after rituximab treatment was also observed in synovial tissue [27,32,33]. Teng and colleagues [33] showed that 88% of RA patients had a reduction of B cells in synovium four weeks after treatment and that clinical responders had less infiltration of CD20^+ ^and CD138^+ ^cells as compared with poor responders [27,33]. Kavanaugh and colleagues [28] also showed that in 80% of RA patients B cell numbers decreased in synovial tissue eight weeks after rituximab treatment [27,28]. Roll and colleagues showed that repopulation of B cells into PB started with B cells expressing CD38 and IgD surface markers, while CD27^+ ^memory B cells repopulated circulation with a significant delay [30]. Similar pattern of B cell regeneration after rituximab treatment was observed in patients with lymphoma and after autologous stem cell transplantation [29,34]. Leandro and colleagues described a depletion of mature BM B cells three months after rituximab treatment, while pro- and pre-B cells as well as immature B cell population and plasma cells were unaffected in BM; however, no baseline samples were obtained [31]. Teng and colleagues investigated the effect of rituximab in BM and concluded that only 8 of 25 patients with RA showed complete depletion of CD19^+ ^B cells, and no phenotypic data were included [33].

In the present study we used serial samples of BM and PB to prospectively follow the ontogeny of B cells shortly after rituximab treatment and distantly, prior to the follow-up of rituximab treatment. We show that rituximab achieves a depletion of IgD^+ ^B cells shortly after the treatment followed by a long-term accumulation of pre-germinal center subsets of B cells in PB combined with a reduction in switched memory B cells both in PB and in BM. We showed that the reduction of switched memory B cells (CD27^+^IgD^-^) does not prevent repopulation with autoantibody-producing B cell clones.

## Materials and methods

### Patients

Thirty-seven patients with established RA diagnosed using the American College of Rheumatology criteria [35], were treated with rituximab (monoclonal anti-CD20 antibodies, Mabthera, Hoffman-La Roche Ltd, Basel, Switzerland) at the Rheumatology Clinic at Sahlgrenska University Hospital, Göteborg, Sweden, between January 2007 and May 2008. Table 1 presents clinical and demographic characteristics of the patients and their immunosuppressive treatment. All patients had been treated with TNFα targeting antibodies prior to rituximab. The anti-TNFα treatment was discontinued at least eight weeks before rituximab treatment. During and after rituximab treatment all the patients were on stable-dose NSAID and DMARDs. Rituximab was provided intravenously in two doses of 1000 mg each on days 1 and 15. The efficacy of rituximab treatment was assessed clinically by disease activity score (DAS) 28, a composite measure based on 28 tender and swollen joint counts, and erythrocyte sedimentation rate. The response to rituximab treatment was evaluated on the basis of European League of Associations for Rheumatology response criteria [36]. The reduction in DAS28 equal to or above 1.2 during the first six months following rituximab treatment was set as the cut-off limit for clinical response. The decision to re-treat with rituximab was based on an increase of clinical disease activity in combination with a patient's wish to be treated. The Ethical Committee at the Sahlgrenska Academy at University of Gothenburg approved this study. All patients gave their written informed consent to participate in the study.

**Table 1:** Clinical and demographic characteristics of patients with rheumatoid arthritis

	RA patients n = 37
Age, years	53 ± 10
(range)	(28-76 years)
Sex, male/female	7/30
Radiological data, erosive/non-erosive	35/2
Rheumatoid factor, +/-	33/4
Duration of the disease, years ± SD	8 ± 6
Treatment	
Methotrexate/other	35/2*
Previous anti-TNF, yes/no	37/0
Previous anti-CD20, yes/no	13/24**
Time after previous anti-CD20, month	22 ± 11 (6-61 months)

* other, 1 chlorambucil, 1 azatioprin.

** One patient is included in both groups i.e. started as non-treated, then returned and was included as treated.

Values are given as mean ± standard deviation (SD). RA = rheumatoid arthritis.

### Collection of blood and BM samples

Heparinized blood and BM aspirates of a volume of 10 ml each were obtained at baseline (n = 37). Blood and BM sampling was repeated one month (weeks 4 to 6; n = 10) and three months (weeks 10 to 14; n = 14) after the first rituximab infusion. PB and BM mononuclear cells were isolated by density gradient separation on Lymphoprep (Axis-Shield PoC As, Oslo, Norway).

### Flow cytometry

The cells were prepared and stained for the Fluorescent Activated Cell Sorting (FACS) analysis as previously described [37,38]. The non-specific binding was blocked with 0.1% rabbit serum. The cells were incubated with dye-conjugated monoclonal antibodies (mAbs), washed, resuspended in FACS-buffer (containing PBS, 1% FCS, 0,1% NaAz and 0.5 mM EDTA), and submitted to five-colour flow cytometry. From each sample 1 × 10^6^–1.5 × 10^7 ^lymphocytes were collected in a FACS Canto II equipped with FACS Diva software (BD-Bioscience, Erebodegem, Belgium). The cells were gated based on the fluorochrome minus one settings when needed [39]. All analyses were performed using the FlowJo software (Three Star Inc., Ashland, OR, USA).

The following monoclonal antibodies were used: anti-CD3 (SK7 or 3K7), anti-CD10 (HI10a), anti-CD19 (HIB19), anti-CD24 (ML5), anti-CD27 (LI28), anti-CD38 (HB7) and anti-CD138 (MI15). All the antibodies were purchased from BD-Bioscience (Erebodegem, Belgium) except for anti-CD19, which were purchased from eBioscience (San Diego, CA, USA). For the Ig analyses we used anti-IgA (F0057), anti-IgD (F0059), anti-IgG (F0056) and anti-IgM (F0058) antibodies (DakoCytomation, Glostrup, Denmark). Polyclonal rabbit F(ab')_2 _anti-human Ig was used as isotype control.

### Phenotype analysis of B cell populations

B cells were defined as CD19^+^CD3^-^. CD27 was used as a memory B cell marker, alone or in combination with IgA, IgD, IgG, and IgM. Combination of CD27 and IgD rendered four different populations: IgD^-^CD27^- ^(immature B cells), IgD^+^CD27^- ^(naïve B cells), IgD^+^CD27^+ ^(unswitched memory B cells), and IgD^-^CD27^+ ^(switched memory B cells and plasma blasts/cells) [40,41]. The maturation level of the B cell populations was determined using a combination of CD38, CD24, and IgD: CD38^++^CD24^++^IgD^+/- ^(immature, transitional, T1), CD38^+^IgD^+^IgM^++^CD24^+^CD27^- ^(mature naive Bm2), CD38^+^IgD^-^CD24^-^CD27^+ ^(mature Bm5), and CD38^+++^IgD^-^D27^+ ^(plasma blasts/cells) [11,42-44]. The first two populations define pre-germinal center B cells, while the last populations consists of post-germinal center B cells.

The mature B cell population (Bm2) is phenotypically close or identical to the naïve B cell population (CD27^-^IgD^+^). To gain more information about immature, pre/pro B cells as well as transitional and germinal center B cell populations, expression of CD10 was also used in combination with CD38 and CD24. Plasma cells were defined as CD138^+^.

### Immunoglobulin secretion

Secretion of Ig was detected using the enzyme-linked immunosorbent spot (ELISPOT) as described [45]. In short, a 96-well nitrocellulose filter plate (Multiscreen, Millipore, Molsheim, France) was coated with 10 μg/ml goat F(ab')_2 _anti-human Ig (Southern Biotech, Birmingham, Alabama, USA). Following blocking, BM and PB mononuclear cells were seed in concentrations 1 × 10^5^, 2 × 10^4^, 4 × 10^3^, and 8 × 10^2 ^lymphocytes per well and incubated for 12 hours. Secreted Ig were detected using goat anti-human antibodies against IgG, IgA, and IgM (Sigma-Aldrich, St Louis, Missouri, USA). Each spot corresponds to one Ig-secreting B cell. RF of Ig-classes G, A, and M was measured in serum samples diluted 1/100 by an ELISA (Hycor Biomedical Ltd, Penicuik, Midlothian, UK). Total level of Igs were analysed nephelometrically.

### Statistical analyses

Statistical analysis of changes in the consequent series of samples obtained the same patient was analysed using the paired t-test. For the analysis of the long-term changes the Mann-Whitney test was used. The *P *value less than 0.05 was considered as significant. All statistical analysis was performed using the GraphPad software Prism (GraphPad Software, San Diego, CA, USA).

## Results

### Short-term effects of rituximab treatment

#### Characteristics of RA patients prior to and following rituximab treatment

Changes in PB and BM leukocyte populations, Ig, and RF at baseline and following rituximab treatment are presented in Table 2. At baseline, all the patients had B cells defined as CD19^+^CD3^- ^cells in PB and BM. One and three months after rituximab treatment, CD19^+^CD3^- ^cells were totally eliminated from the PB of all but one patient. In contrast, BM from the same patients analysed at the same time points retained up to 30% of B cells, which gave a possibility to follow the ontogeny of B cells in the paired samples of BM obtained prior and shortly after rituximab treatment.

**Table 2:** Serological characteristics of rheumatoid arthirits patients prior to and following rituximab treatment

	**Bone marrow**			**Peripheral blood**	
	
	Baseline	1 month	3 months	Baseline	3 months
**WBC, 10^6^/ml**	20.5 ± 12.6	17.5 ± 6.5	24.2 ± 15.9	6.8 ± 3.0	7.1 ± 3.2
**CD19+, %**	5.6 ± 3.7	1.1 ± 0.9**	1.6 ± 1.3**	12.2 ± 6.4	0***
**CD3+, %**	28.3 ± 12.7	36.2 ± 14.3	22.7 ± 6.2	46.9 ± 16.2	42.8 ± 13.9
**CD138+, %**	0.83 ± 0.56	0.70 ± 0.47	0.73 ± 0.51	-	-
**ELISPOT, 10^6 ^lymphocytes/ml**					
IgG	11073 ± 11363	10574 ± 8414	7703 ± 7451	1504 ± 3383	427 ± 800
IgM	8163 ± 8448	5922 ± 5492**	4058 ± 6833	2013 ± 7070	123 ± 223
IgA	6009 ± 4628	7010 ± 6345	5122 ± 4015	1280 ± 2743	471 ± 1094
**RF, U/ml**					
IgG	Not assessed	Not assessed	Not assessed	50 ± 31	34 ± 26***
IgM	Not assessed	Not assessed	Not assessed	78 ± 31	64 ± 49**
IgA	Not assessed	Not assessed	Not assessed	37 ± 28	30 ± 25**
**Total Igs, mg/L**					
IgG	Not assessed	Not assessed	Not assessed	13 ± 4.5	12.3 ± 4.2***
IgM	Not assessed	Not assessed	Not assessed	1.9 ± 1.2	1.6 ± 1.1**
IgA	Not assessed	Not assessed	Not assessed	3.6 ± 1.8	3.0 ± 1.3

* *P *≤ 0.05; ** *P *≤ 0.01; *** *P *≤ 0.001.

Values are given as mean ± standard deviation.

Ig = immunoglobulin; RF = rheumatoid factor; WBC = white blood cell.

**Figure 1:**
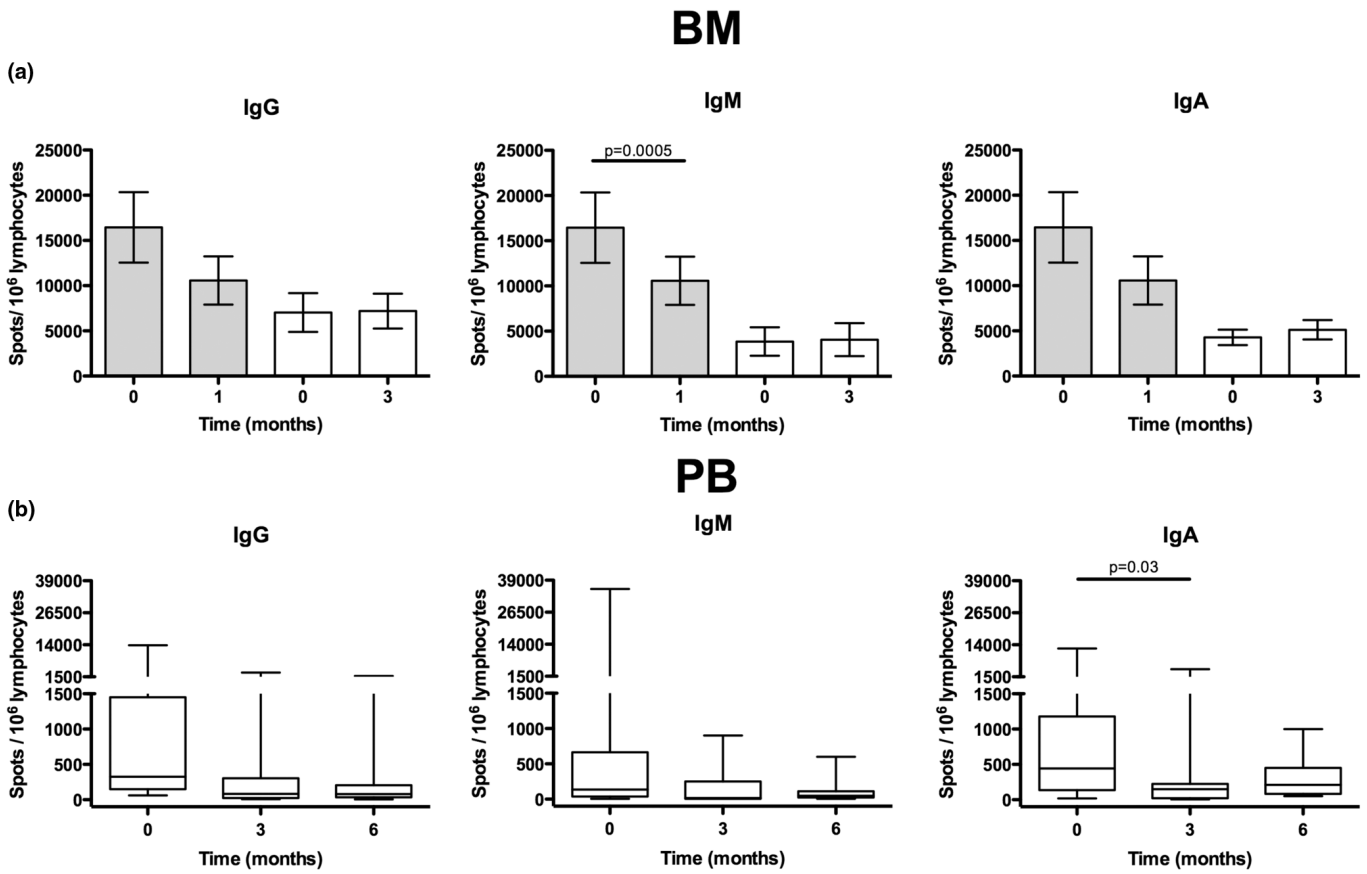
Short-term changes of the Ig-secreting cells in BM and PB after rituximab treatment. **(a) **Number of immunoglobulin (Ig)-secreting cells in bone marrow (BM) isolated from patients with rheumatoid arthritis at day 0, 1 and 3 months after rituximab treatment. Paired with respect to the sampling occasion. Error bars respresenting mean ± standard error of the mean. **(b) **Ig-secreting cells in peripheral blood (PB) at day 0, 3 and 6 months after rituximab treatment. Box represents 25^th ^to 75^th ^percentile, line indicates median, whereas error bars represent range. Statistical evaluation was performed using paired t-test.

**Figure 2:**
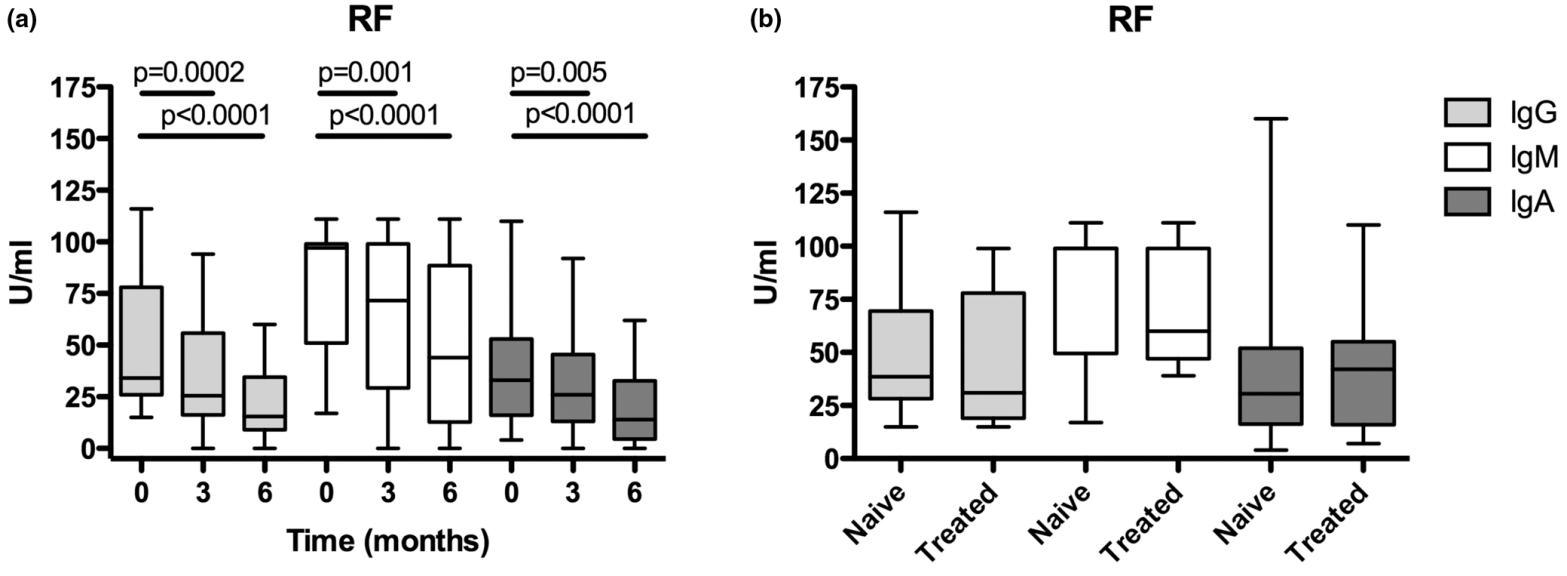
Short- and long-term changes of RF levels in PB after rituximab treatment. **(a) **Rheumatoid factor (RF)-levels in peripheral blood (PB) at day 0, 3 and 6 months after rituximab treatment. **(b) **RF-levels in PB comparing rituximab-naïve and treated patients. Box represents 25^th ^to 75^th ^percentile, line indicates median, whereas error bars represent range. Statistical evaluation was performed using paired t-test (short-term changes) and Mann-Whitney t-test (long-term changes).

Evaluation of Ig secretion in BM using ELISPOT one to three months after rituximab treatment showed a significant decrease of IgM producing cells after one month (*P *= 0.0005; Figure 1a). The secretion of IgA and IgG in BM was unchanged at all time points. In contrast, a significant decrease of IgA-producing cells (*P *= 0.03) was observed in PB after three months (Figure 1b). The levels of autoreactive antibodies (RF of IgG, IgM and IgA isotypes) in PB were reduced by approximately 50% (Figure 2a), while the total levels of circulating Igs were unchanged (not shown).

**Figure 3:**
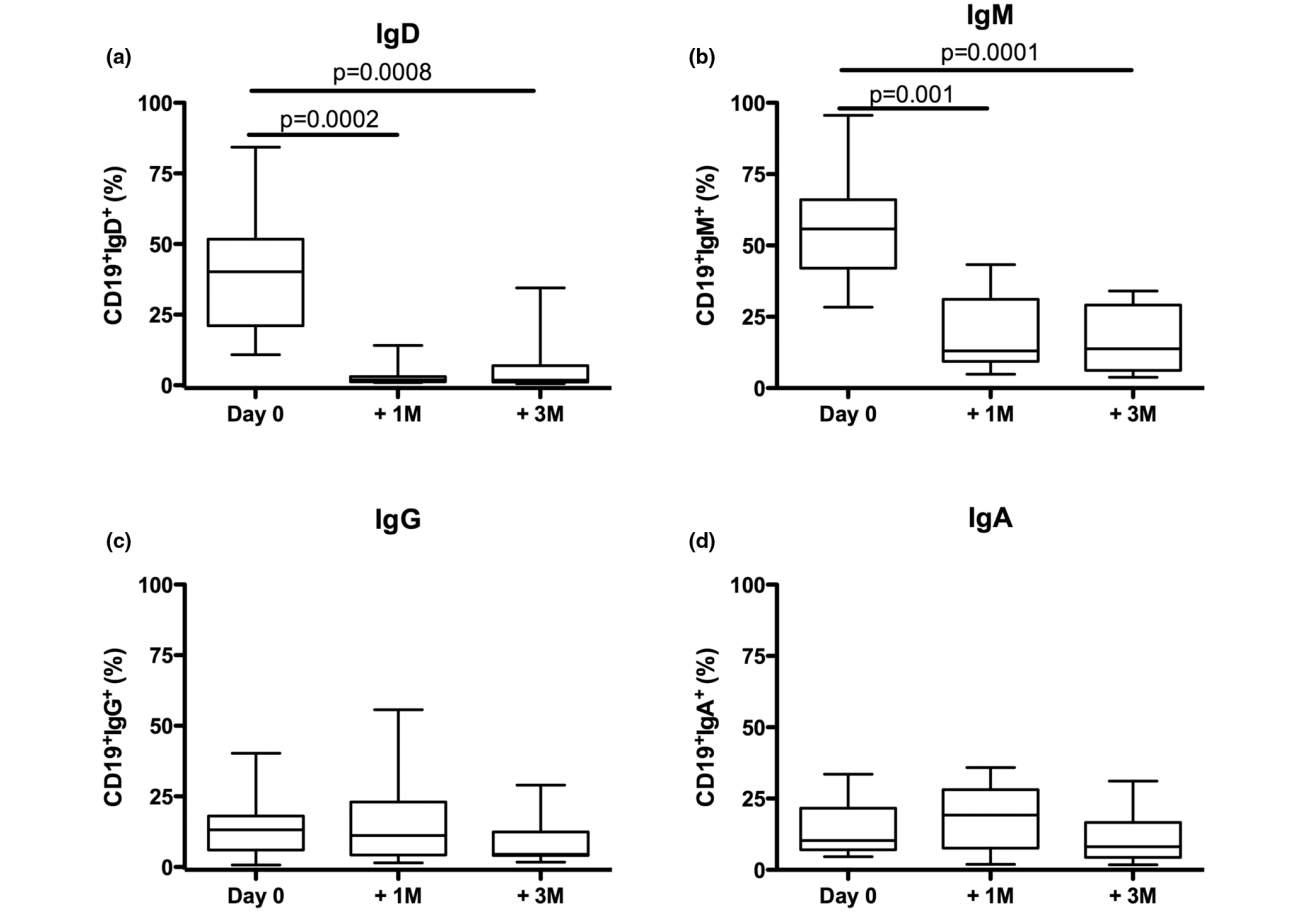
Short-term changes in the immunoglobulin expression of B cells following rituximab treatment. Isolated bone marrow mononuclear cells were stained for immunoglobulin (Ig) expression at day 0, 1 and 3 months after rituximab treatment. In **(a) **CD19^+^IgD^+^, **(b) **CD19^+^IgM^+^, **(c) **CD19^+^IgG^+ ^and **(d) **CD19^+^IgA^+ ^is shown. Box represents 25^th ^to 75^th ^percentile, line indicates median, whereas error bars represent range. Statistical evaluation was performed using paired t-test.

Analysis of Ig expression on BM B cells using flow cytometry one month (n = 10) and three months (n = 14) after rituximab treatment revealed a pronounced decrease in frequency of IgD^+ ^as well as IgM^+ ^(Figure 3). In contrast, the proportion of CD19^+^CD3^- ^cells expressing surface IgA and IgG remained unchanged (Figure 3).

#### Rituximab depletes immature and naïve B cells in BM

**Figure 4:**
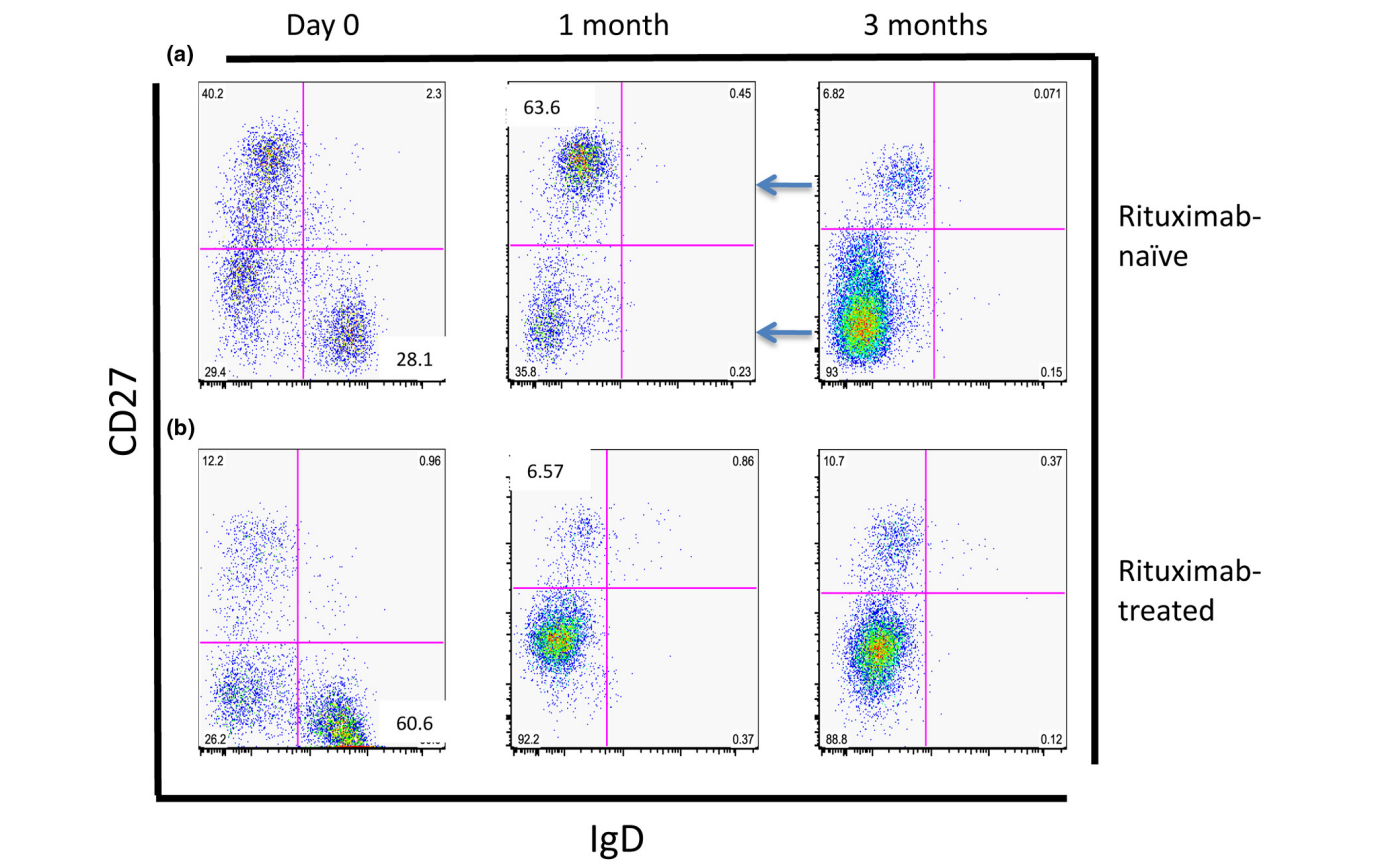
A representative plot of short-term and long-term changes of the B cell expression of CD27 and IgD on B cells in bone marrow from patients with rheumatoid arthritis receiving rituximab treatment. Lower left quadrant = immature B cells (IgD^-^CD27^-^); lower right quadrant = naïve B cells (IgD^+^CD27^-^); upper right quadrant = unswitched memory B cells (IgD^+^CD27^+^); and upper left quadrant = switched memory B cells (IgD^-^CD27^+^). **(a) **Rituximab-naïve patient is shown at day 0, 1 month and 3 months following treatment. **(b) **Rituximab-treated patient is shown at day 0, 1 month and 3 months following treatment. Arrow indicates depleted populations.

To further evaluate the phenotype of B cells escaping rituximab depletion in BM, a combination of CD27 and IgD was used. A representative dot plot is shown in Figure 4. Cumulative results of B cell populations in absolute numbers are given in Table 3. We found a pronounced depletion of naïve B cells (CD27^-^IgD^+^) after one and three months (*P *= 0.0007, and *P *< 0.0001). Furthermore, a reduction of immature B cells (CD27^-^IgD^-^; *P *= 0.005) and unswitched B cells (CD27^+^IgD^+^) after three months (*P *= 0.02), and switched memory B cells (CD27^+^IgD^-^; *P *= 0.01) after one month was also detected. Importantly, almost all of the B cell populations decreased when analysing absolute numbers as shown in Table 3. The majority of the surviving B cells was found within the IgD^- ^populations. This argues for a predominant depletion of IgD^+ ^B cells consisting of the naïve and unswitched B cell population. In contrast, switched memory B cells escape depletion despite their surface expression of CD20.

**Table 3:** Absolute numbers of B cells in bone marrow (per 10^6 ^mononuclear cells)

	**CD27^-^IgD^- ^(Immature)**		**CD27^-^IgD^+ ^(Naïve)**		**CD27^+^IgD^+ ^(Unswitched)**		**CD27^+^IgD^- ^(Switched)**	
	
	n = 10	n = 14	n = 10	n = 14	n = 10	n = 14	n = 10	n = 14
**Baseline**								
Day 0	134 ± 100	282 ± 315	165 ± 158	213 ± 116	17 ± 39	29 ± 38	70 ± 48	161 ± 105
**Short-term**								
1 month	53 ± 75 *P *= 0.004		4 ± 4 *P *= 0.002		0.5 ± 0.7 *P *= 0.004		40 ± 32 *P *= 0.01	
3 months		98 ± 114 *P *= ns		5 ± 9 *P *= 0.0002		2 ± 4 *P *= 0.0002		39 ± 31 *P *= 0.0006
Post-RTX survival %	40	35	2	2	3	7	57	24

* Values are given as mean ± standard deviation.

** Statistics are calculated with paired T-test, *P*-values are given in comparison to day 0.

RTX = rituximab.

#### Rituximab treatment results in a total depletion of CD38 expressing B cells in BM

**Figure 5:**
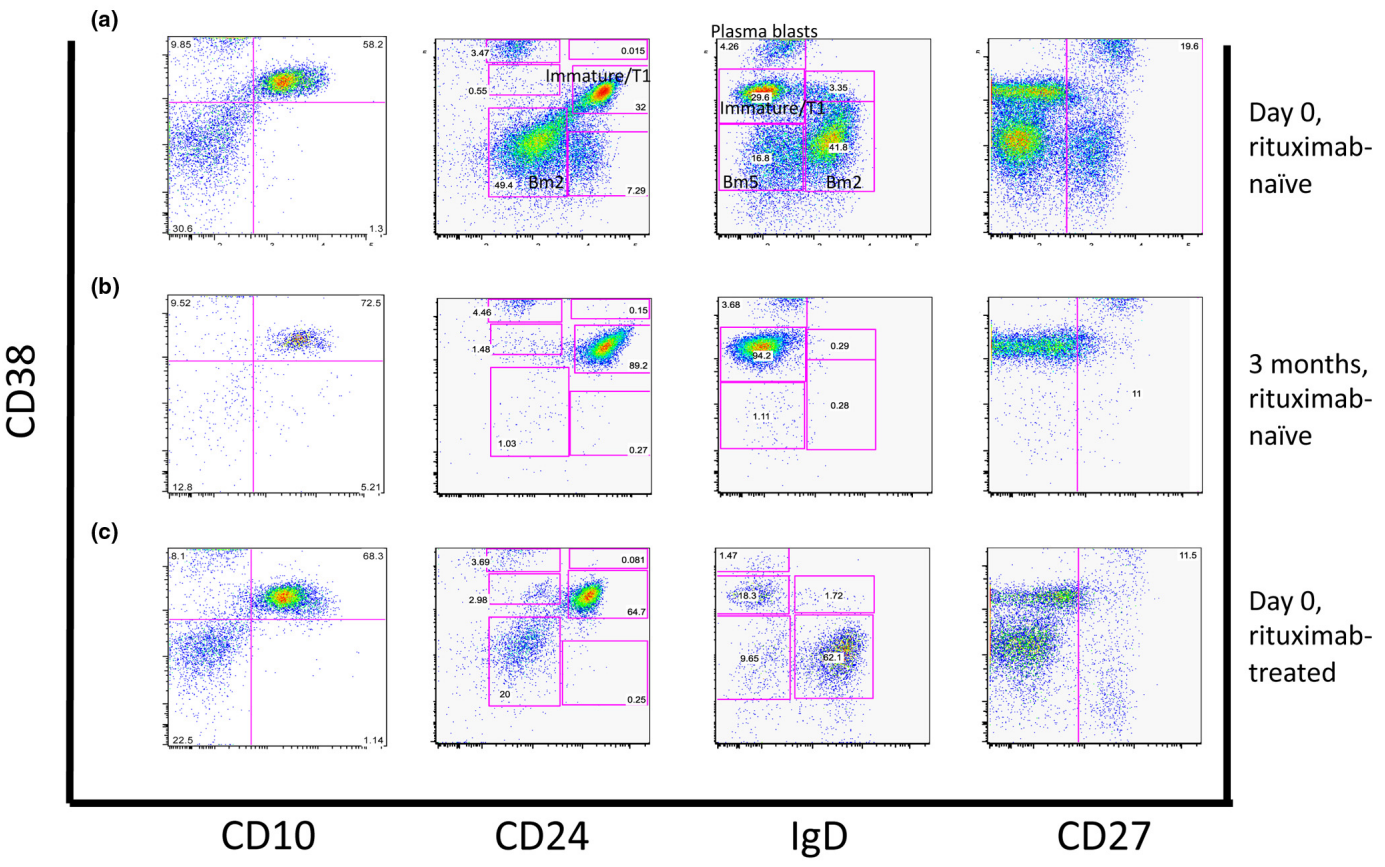
A representative plot of short-term and long-term changes of the B cell expression of CD38 in combination with CD10/CD24/IgD or CD27 in BM from RA patients receiving rituximab treatment. B cells expressing CD38 were analysed with respect to CD10/CD24, IgD or CD27 expression. Using CD38/IgD, plasmablast (CD38^+++^IgD^-^), immature and transitional B cells (CD38^++^IgD^+/-^), Bm5 (CD38^+^IgD^-^), Bm2 (CD38^+^IgD^+^) populations were defined. B cells from a rituximab-naïve patient at **(a) **day 0, **(b) **after 3 months, and **(c) **in a rituximab-treated patient at day 0, is shown for the combination of CD38/CD10/CD24/IgD/CD27.

The expression of CD38 in combination with IgD was analysed for further characteristics of B cell maturation in BM shortly after rituximab treatment. A representative dot plot is shown in Figure 5. The absolute numbers of B cells in the defined populations are shown in Table 4. We found a significant reduction of mature Bm2 (CD38^+^IgD^+^; *P *= 0.0007, *P *< 0.0001, at one and three months, respectively) and of Bm5 (CD38^+^IgD^-^; *P *= 0.02, at one month) B cells. The population of immature and transitional (CD38^++^IgD^-^) B cells as well as the plasma blasts (CD38^+++^IgD^-^) were not depleted by rituximab treatment. To ascertain the low maturation status of the immature B cells a combination of CD38, CD24, and CD10 was used. The frequency of expression of CD24/CD10 was clearly increased within the remaining B cell population (Figure 5). The analysis of B cells with respect to CD38 expression shows a predominant depletion of Bm5 and mature Bm2. As high expression of CD38 may be characteristic for plasma cells, defined here as CD138^+^, we analysed the precursors of plasma cells in BM before and after rituximab treatment. No significant changes in plasma cell numbers were observed following rituximab treatment indicating that plasma cells are not affected by rituximab (Table 2).

**Table 4:** Absolute numbers of B cells in bone marrow (per 10^6 ^mononuclear cells)

	**CD38+IgD-** **(Bm5)**		**CD38+IgD+** **(Mature Bm2)**		**CD38++IgD-** **(Immature/T1)**		**CD38++IgD+** **(Immature/T1)**		**CD38+++IgD-** **(Plasma blasts)**	
	
	N = 10	n = 14	n = 10	n = 14	n = 10	n = 14	n = 10	n = 14	n = 10	n = 14
**Baseline**										
Day 0	34 ± 32	55 ± 51	140 ± 135	203 ± 140	96 ± 98	180 ± 289	19 ± 15	35 ± 49	27 ± 16	38 ± 31
**Short-term**										
1 month	2 ± 2 *P *= 0.01		0.9 ± 1 *P *= 0.01		48 ± 71 *P *= ns		9 ± 25 *P *= ns		21 ± 19 *P *= ns	
3 months		12 ± 32 *P *= 0.04		1 ± 2 *P *= 0.0001		86 ± 97 *P *= ns		3 ± 5 *P *= 0.03		23 ± 29 *P *= ns
Post-RTX survival %	6	22	1	0.5	50	48	47	9	78	61

* Values are given as mean ± standard deviation.

** Statistics are calculated with paired T-test, *P*-values are given in comparison to day 0.

RTX = rituximab.

### Long-term effects of rituximab treatment

**Figure 6:**
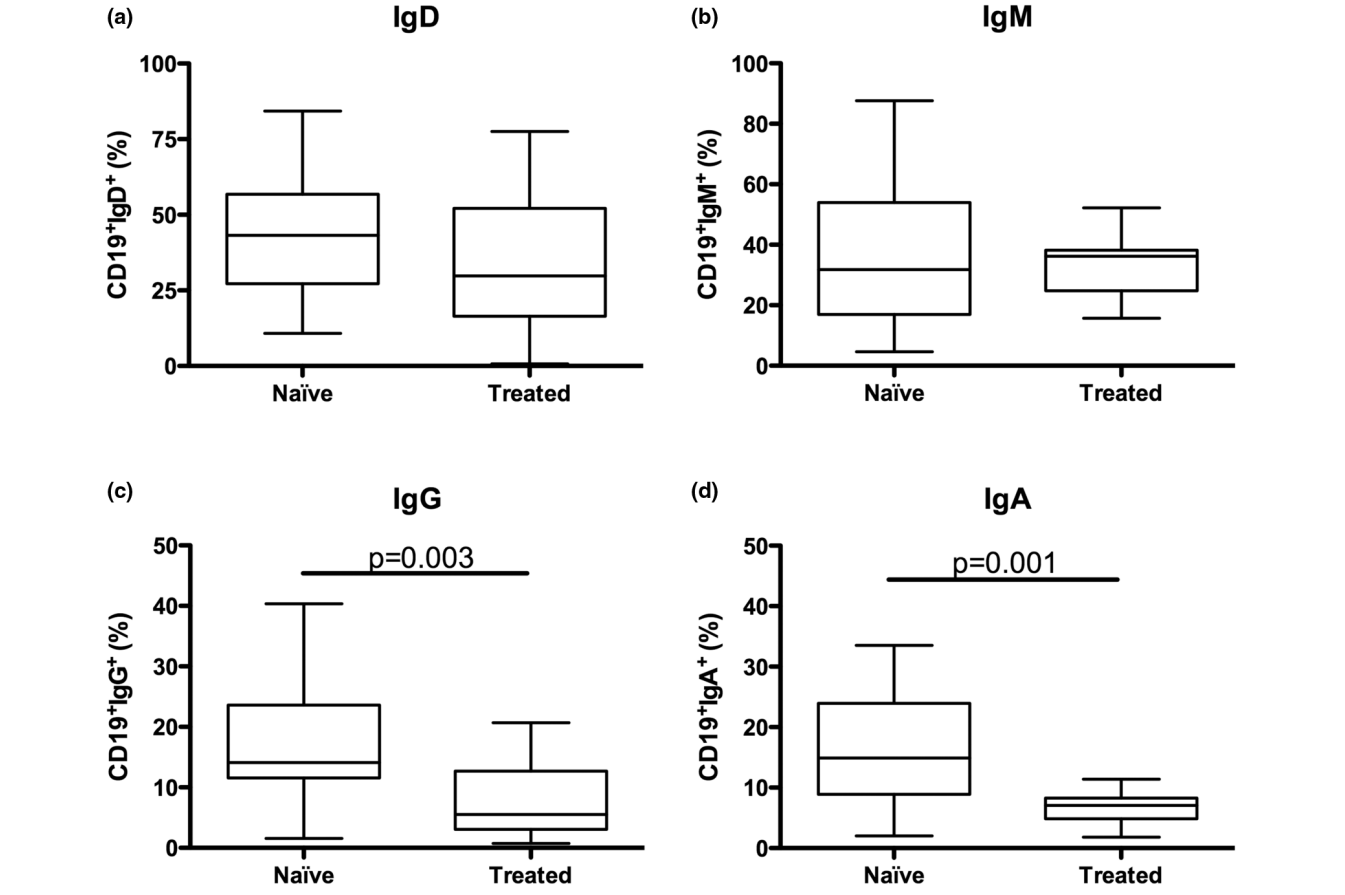
Long-term changes in the immunoglobulin expression of B cells following rituximab treatment. Isolated bone marrow mononuclear cells were stained for immunoglobulin (Ig) expression comparing rituximab-naïve and treated patients. In **(a) **CD19^+^IgD^+^, **(b) **CD19^+^IgM^+^, **(c) **CD19^+^IgG^+ ^and **(d) **CD19^+^IgA^+ ^is shown. Box represents 25^th ^to 75^th ^percentile, line indicates median, whereas error bars represent range. Statistical evaluation was performed using the Mann-Whitney t-test.

To evaluate long-term effects of rituximab, we divided the patients into two groups: those who were not treated with rituximab previously, referred to as rituximab-naïve (n = 24), and those who had been treated with rituximab previously (mean 22 months, range 6 to 61 months) referred to as rituximab-treated patients (n = 13). At admission, these two groups of patients were similar with respect to activity RA (DAS28: 6.00 ± 0.76 vs. 5.64 ± 0.58, respectively) and the number of B cells in PB and BM (13 ± 5% vs. 11 ± 4%). Analysing the expression of surface-Ig on CD19^+ ^BM mononuclear cells showed a decreased frequency of IgG and IgA (*P *= 0.003, *P *= 0.001) in rituximab-treated patients as compared with rituximab-naïve patients (Figure 6). No differences between the groups were found regarding the expression of IgD and IgM (Figure 6). BM from rituximab-treated patients displayed a decrease of IgM-secreting cells as compared with rituximab-naïve patients, while in PB the levels of Ig-producing cells were similar (Figure 1a). The levels of total Ig levels as well as the circulating RF (Figure 2b) were similar between the rituximab-naïve and rituximab-treated groups.

#### Decreased proportion of CD27^+ ^memory B cells in BM and is a hallmark of rituximab treatment

The analysis of CD27 expression in BM showed that rituximab-treated patients had a significantly lower proportion of CD27^+ ^memory B cells (*P *= 0.0004) compared with those who were rituximab naïve (data not shown). This was consequently followed by a reduction in the unswitched (CD27^+^IgD^+^, *P *< 0.0001) as well as in the switched memory cells (CD27^+^IgD^-^, *P *= 0.004) in BM, and by an increase of immature (CD27^-^IgD^-^) B cells (*P *= 0.01). The absolute numbers of B cells in the rituximab-treated and tituximab-naïve patients are shown in Table 5. No correlation was found between the time elapsed after previous rituximab treatment and the amount of immature (CD27^-^IgD^-^) B cells in BM.

**Table 5:** Absolute numbers of B cells in bone marrow (per 10^6 ^mononuclear cells)

**Long-term**	**CD27^-^IgD^-^** **(Immature)**	**CD27^-^IgD^+^** **(Naïve)**	**CD27^+^IgD^+^** **(Unswitched)**	**CD27^+^IgD^-^** **(Switched)**	**Total number of CD19+ cells**
RTX-naïve n = 24	185 ± 247 (32%)	206 ± 134 (36%)	35 ± 44 (6%)	148 ± 94 (26%)	574 ± 129 (100%)
RTX-treated n = 13	260 ± 251 (51%) *P *= ns	174 ± 147 (34%) *P *= ns	4 ± 3 (1%) *P *= 0.0009	68 ± 72 (13%) *P *= 0.001	506 ± 118 (100%)

* Values are given as mean ± standard deviation.

** Statistics are calculated with paired T-test, *P*-values are given in comparison to day 0.

RTX = rituximab.

#### Accumulation of immature subset of B cells in BM long after rituximab treatment

We found a proportional increase of immature and transitional (CD38^++^IgD^-^, *P *= 0.002) and a reduction of Bm5 cells (CD38^+^IgD^-^, *P *< 0.0001) in rituximab-treated patients as compared with rituximab-naïve. The absolute numbers of B cells in the rituximab-treated and rituximab-naïve patients are shown in Table 6. The accumulation of immature subset of B cells in BM of rituximab-treated patients was proved by a prevalence of CD24 expression in immature transitional B cell populations. These findings support our observation on the accumulation of pre-germinal center B cells long after rituximab treatment.

**Table 6:** Absolute numbers of B cells in bone marrow (per 10^6 ^mononuclear cells)

**Long-term**	**CD38+IgD-** **(Bm5)**	**CD38+IgD+** **(Mature Bm2)**	**CD38++IgD-** **(Immature/T1)**	**CD38++IgD+** **(Immature/T1)**	**CD38+++IgD-** **(Plasma blasts)**
RTX-naïve n = 24	75 ± 54	205 ± 139	171 ± 258	39 ± 51	35 ± 26
RTX-treated n = 13	17 ± 19 *P *= 0.0004	155 ± 134 *P *= ns	222 ± 292 *P *= ns	19 ± 15 *P *= ns	22 ± 18 *P *= ns

* Values are given as mean ± standard deviation.

** Statistics are calculated with paired T-test, *P*-values are given in comparison to day 0.

RTX = rituximab.

## Discussion

In the present study we analysed consequences of rituximab treatment on the ontogeny of B cells in BM and in PB shortly after and prior to follow-up rituximab treatment. The short-term changes were characterised by a depletion of naïve and unswitched memory B cells (IgD^+^) as well as CD38^+ ^populations including mature Bm2 (CD38^+^IgD^+^) and Bm5 B cells (CD38^+^IgD^-^). The long-term changes were characterized by a decrease of the memory B cell population in BM.

The evaluation of B cell populations using CD38 marker showed that the switched memory B cells (CD27^+^IgD^-^) were preserved in BM while the pre-germinal center population (Bm2, T1) of B cells were depleted. The short-term changes were characterised by a total depletion of IgD^+^CD38^+ ^B cells in BM. The remaining BM B cell population consists of CD27^-^IgD^- ^immature B cells, and mostly CD27^+^IgD^- ^switched memory B cells. Simultaneously, the levels of RF and Ig-secreting cells in circulation are decreased by 50% three to six months after rituximab treatment. These data suggest that IgD^+^CD38^+ ^B cell population or IgM expressing B cell population may be responsible for production of autoreactive Igs. Similar data in PB are also shown by Koelsch and colleagues [46].

Our findings indicate that switched memory B cells are better survivors of rituximab despite the expected surface expression of CD20. The properties of B cells leading to rituximab resistance and helping 30% of human BM B cells to escape depletion are elucidated. Similar results were obtained by Teng and colleagues who also showed that rituximab did not achieve a complete depletion of B cells in BM [33]. One of the possible explanations is a lack of or low intensity of CD20 expression on the surface of B cells. Indeed, many B cell precursors and late-stage differentiated B cells (i.e. some plasma blasts/cells) lack CD20 but may express CD19 making them unresponsive to rituximab treatment. We defined B cell population as CD19^+^, thus discrepancy between CD20 and CD19 expression is difficult to address in our study. It has been shown in animal experiments that the remaining B cells preserved in circulation following rituximab treatment may be memory B cells [26,47,48]. Several studies have shown that mature B cells can escape depletion even though they express CD20 [49-51]. Another suggested mechanism protecting B cells from depletion with rituximab is the expression of high levels of CD38 and a simultaneous lack of IgD [52-54]. CD38 expressing cells possibly have low levels or a lack of CD20 and this may be a reason for their survival in bone marrow [52-54].

In our group of patients, we used a combination of CD38 and IgD, as a complement to the analysis of CD27 and IgD, to ascertain the maturity stage of B cells and to closer define the B cell population depleted by rituximab. Both ways of B cell analyses show that IgD^- ^population is better preserved after rituximab therapy.

We also showed that the levels of RF are strongly reduced following rituximab treatment, while the total levels of total Igs in circulation remain stable, suggesting: a selective depletion; a depletion of a 'more naïve' B cell population; or a depletion of B cell population potentially responsible for autoantibody secretion.

The long-term follow-up of rituximab effects shows no differences regarding the levels of circulating RFs and Igs in the rituximab-naïve and rituximab-treated patients. This suggests that autoreactive clones of B cells are only temporarily depleted by rituximab while the precursors of autoreactive B cell clones in BM as well as the cells providing signals triggering their development remain unaffected by rituximab. The return of RF into circulation occurred in parallel to the repopulation of naïve (IgD^+^CD27^-^) as well as IgM^+^CD27^- ^B cells into BM and PB of RA patients admitted for the next course of rituximab treatment. This supports the theory that these B cells may be autoreactive [46]. During the evaluation of distant effects of rituximab, we observed that the development of naïve mature B cells from immature and transitional B cells (CD38^++^IgD^-^) remained unaffected. The reduced levels of memory B cells were probably caused by a reduction of post-germinal center Bm5 (CD38^+^IgD^-^) in PB. One of the explanations for this may be a normal development of immature B cells in BM and an inability of naïve (CD27^-^) B cells to enter peripheral lymphoid organs or germinal centers resulting in their accumulation in PB [30,55]. Our study is limited to B cell development in the BM, thus we may only speculate about B cell maturation outside the BM, namely in lymph nodes and in germinal centers. Physiological consequences of the inability to develop memory cells long after rituximab treatment need further evaluation primarily with respect to changes in antigen presentation and humoral immune responses in RA patients treated with repeated courses of rituximab.

## Conclusions

To conclude, rituximab achieves a depletion of naïve and unswitched B cell populations shortly after the treatment, which is followed by a long-term reduction in switched memory B cells both in PB and in BM. The reduction of switched memory B cells does not prevent repopulation with autoantibody producing B cell clones suggesting that mechanisms regulating the formation of autoreactive clones are not disrupted by rituximab.

## Abbreviations

BM: bone marrow; DAS28: disease activity score; DMARD: disease-modifying antirheumatic drug; ELISA: enzyme-linked immunosorbent assay; ELISPOT: enzyme-linked immunosorbent spot; FACS: Fluorescent Activated Cell Sorting; Ig: immunoglobulins; NSAID: non-steroidal anti-inflammatory drug; PB: peripheral blood; RA: rheumatoid arthritis; RF: rheumatoid factor; TNF: tumor necrosis factor.

## Competing interests

The authors declare that they have no competing interests.

## Authors' contributions

MR performed science, analysed data and wrote paper; SA performed science and analysed data; AT planned science; MIB planned science, collected patient material, analysed data, and wrote paper; MB planned science, performed science, analysed data, and wrote paper.
